# 3-(4-Meth­oxy­phen­yl)-6,7-dihydro-1*H*-furo[3,4-*c*]pyran-4(3*H*)-one

**DOI:** 10.1107/S160053681204723X

**Published:** 2012-11-24

**Authors:** Jingyi Zhang, Ye An, Yikai Zhang, Guobing Shi

**Affiliations:** a Department of Pharmacy, General Hospital of Shenyang Military Command, Shenyang 110016, People’s Republic of China; b Department of Medicinal Chemistry, School of Pharmacy, Second Military Medical University, Shanghai 200433, People’s Republic of China

## Abstract

In the title compound, C_14_H_14_O_4_, the dihedral angle between the hydro­furan and benzene rings is 88.41 (15)°. The hydro­pyran ring adopts an envelope conformation, with the O-bound methyl­ene C atom as the flap. In the crystal, weak aromatic π–π stacking is observed [centroid–centroid separation = 3.848 (2) Å].

## Related literature

For medicinal background, see: Wang *et al.* (2011 ▶).
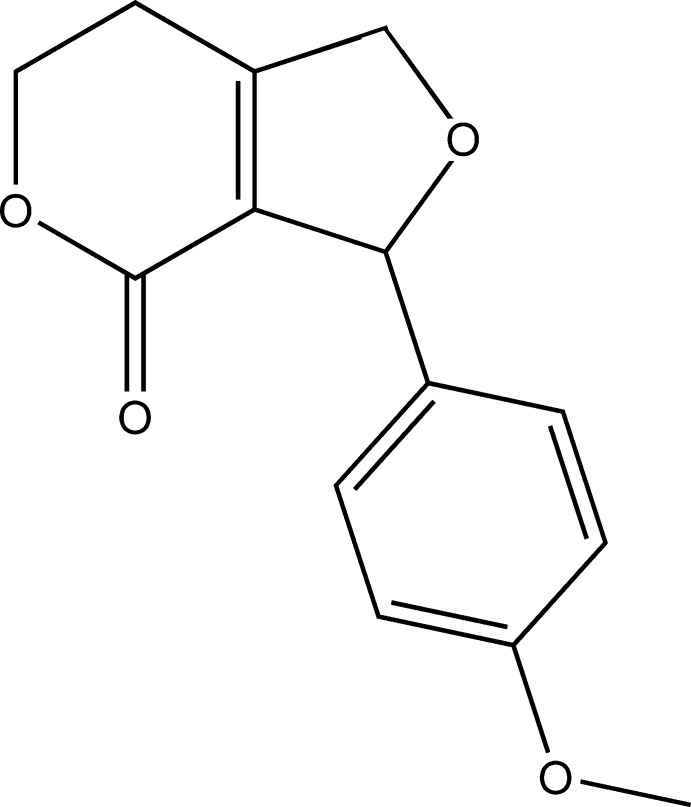


## Experimental

### 

#### Crystal data

C_14_H_14_O_4_
*M* *_r_* = 246.25
Monoclinic, 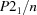

*a* = 7.240 (3) Å
*b* = 8.635 (4) Å
*c* = 19.545 (8) Å
β = 97.352 (6)°
*V* = 1212.0 (9) Å^3^
*Z* = 4
Mo *K*α radiation
μ = 0.10 mm^−1^
*T* = 293 K
0.25 × 0.25 × 0.20 mm

#### Data collection

Bruker SMART CCD diffractometer
Absorption correction: multi-scan (*SADABS*; Bruker, 2002 ▶) *T*_min_ = 0.976, *T*_max_ = 0.981
4872 measured reflections
2127 independent reflections
1494 reflections with *I* > 2σ(*I*)
*R*_int_ = 0.104

#### Refinement

*R*[*F* ^2^ > 2σ(*F* ^2^)] = 0.074
*wR*(*F* ^2^) = 0.227
*S* = 1.06
2127 reflections
164 parameters
6 restraints
H-atom parameters constrained
Δρ_max_ = 0.55 e Å^−3^
Δρ_min_ = −0.31 e Å^−3^

### 

Data collection: *SMART* (Bruker, 2002 ▶); cell refinement: *SAINT* (Bruker, 2002 ▶); data reduction: *SAINT*; program(s) used to solve structure: *SHELXS97* (Sheldrick, 2008 ▶); program(s) used to refine structure: *SHELXL97* (Sheldrick, 2008 ▶); molecular graphics: *SHELXTL* (Sheldrick, 2008 ▶); software used to prepare material for publication: *SHELXTL*.

## Supplementary Material

Crystal structure: contains datablock(s) I, New_Global_Publ_Block. DOI: 10.1107/S160053681204723X/hb6991sup1.cif

Supplementary material file. DOI: 10.1107/S160053681204723X/hb6991Isup2.cdx

Structure factors: contains datablock(s) I. DOI: 10.1107/S160053681204723X/hb6991Isup4.hkl

Supplementary material file. DOI: 10.1107/S160053681204723X/hb6991Isup6.cdx

Additional supplementary materials:  crystallographic information; 3D view; checkCIF report

## Figures and Tables

**Fig. 1.**
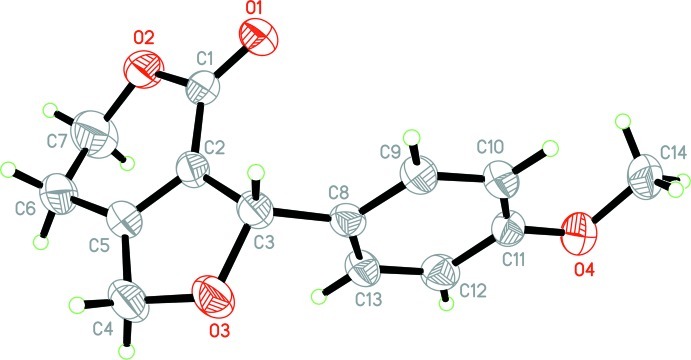
The molecular structure of the title compound, with 30% probability displacement ellipsoids for non-H atoms.

**Fig. 2.**
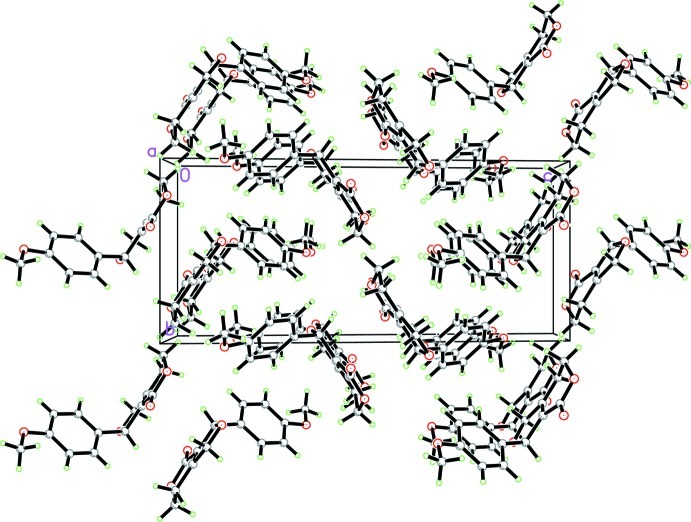
A view of the unit-cell contents for the title compound.

## supplementary crystallographic information

### Experimental

NaH (60% in mineral oil, 24 mmol) was added to a solution of but-2-yne-1,4-diol (2.58 g, 30 mmol) in THF (50 ml) under nitrogen, and the solution was stirred for 5 min at 20 C. Diethyl 2-(4-methoxybenzylidene)malonate (5.56 g, 20 mmol) and CuI (0.38 g, 2 mmol) were then added successively. When consumption of the starting materials was observed by TLC, the reaction mixture was added 3% HCl solution until the PH value was 7. Then the mixture was extracted with CH_2_Cl_2_ (30 ml × 3). The combined organic layers were dried and solids were combined. The solid (0.973 g, 3 mmol) subsequently was reacted with 20% KOH in EtOH/THF (15/15 ml) at room temperature for 6 h. Then the reaction mixture was diluted with CH_2_Cl_2_ (30 ml) and washed with saturated Na_2_CO_3_, brine and dried with MgSO4. The mixture was purified with silica gel column chromagraphy. The Trans-form compounds could be obtained. Yield: 10%. *M*. p.: 407 K.

### Refinement

All hydrogen atoms were placed in calculated positions using a riding model, with d (C—H) = 0.93 Å for aromatic, 0.97 Å for CH2 and 0.96 Å for CH3 groups, and with *U*_iso_ (H) = 1.2 *U*_eq_(C).

### Crystal data

**Table:**

C_14_H_14_O_4_	*F*(000) = 520
*M**_r_* = 246.25	*D*_x_ = 1.350 Mg m^−^^3^
Monoclinic, *P*2_1_/*n*	Mo *K*α radiation, λ = 0.71073 Å
Hall symbol: -P 2yn	Cell parameters from 735 reflections
*a* = 7.240 (3) Å	θ = 2.6–24.3°
*b* = 8.635 (4) Å	µ = 0.10 mm^−^^1^
*c* = 19.545 (8) Å	*T* = 293 K
β = 97.352 (6)°	Prism, colorless
*V* = 1212.0 (9) Å^3^	0.25 × 0.25 × 0.20 mm
*Z* = 4	

### Data collection

**Table:**

Bruker SMART CCD diffractometer	2127 independent reflections
Radiation source: fine-focus sealed tube	1494 reflections with *I* > 2σ(*I*)
Graphite monochromator	*R*_int_ = 0.104
phi and ω scans	θ_max_ = 25.0°, θ_min_ = 2.6°
Absorption correction: multi-scan (*SADABS*; Bruker, 2002)	*h* = −8→7
*T*_min_ = 0.976, *T*_max_ = 0.981	*k* = −10→10
4872 measured reflections	*l* = −23→19

### Refinement

**Table:**

Refinement on *F*^2^	Primary atom site location: structure-invariant direct methods
Least-squares matrix: full	Secondary atom site location: difference Fourier map
*R*[*F*^2^ > 2σ(*F*^2^)] = 0.074	Hydrogen site location: inferred from neighbouring sites
*wR*(*F*^2^) = 0.227	H-atom parameters constrained
*S* = 1.06	*w* = 1/[σ^2^(*F*_o_^2^) + (0.1396*P*)^2^] where *P* = (*F*_o_^2^ + 2*F*_c_^2^)/3
2127 reflections	(Δ/σ)_max_ = 0.001
164 parameters	Δρ_max_ = 0.55 e Å^−^^3^
6 restraints	Δρ_min_ = −0.31 e Å^−^^3^

### Special details

**Table:**

Geometry. All e.s.d.'s (except the e.s.d. in the dihedral angle between two l.s. planes) are estimated using the full covariance matrix. The cell e.s.d.'s are taken into account individually in the estimation of e.s.d.'s in distances, angles and torsion angles; correlations between e.s.d.'s in cell parameters are only used when they are defined by crystal symmetry. An approximate (isotropic) treatment of cell e.s.d.'s is used for estimating e.s.d.'s involving l.s. planes.
Refinement. Refinement of *F*^2^ against ALL reflections. The weighted *R*-factor *wR* and goodness of fit *S* are based on *F*^2^, conventional *R*-factors *R* are based on *F*, with *F* set to zero for negative *F*^2^. The threshold expression of *F*^2^ > σ(*F*^2^) is used only for calculating *R*-factors(gt) *etc*. and is not relevant to the choice of reflections for refinement. *R*-factors based on *F*^2^ are statistically about twice as large as those based on *F*, and *R*- factors based on ALL data will be even larger.

### Fractional atomic coordinates and isotropic or equivalent isotropic displacement parameters (Å^2^)

**Table:**

	*x*	*y*	*z*	*U*_iso_*/*U*_eq_	
O1	0.3995 (3)	0.6361 (2)	0.04179 (10)	0.0677 (6)	
O2	0.5812 (3)	0.8161 (3)	0.00885 (11)	0.0771 (7)	
O3	0.9242 (3)	0.4487 (2)	0.14388 (12)	0.0777 (7)	
O4	0.2905 (3)	0.5087 (3)	0.33985 (12)	0.0800 (7)	
C1	0.5550 (4)	0.6850 (3)	0.04378 (13)	0.0541 (7)	
C2	0.7206 (3)	0.6116 (3)	0.07879 (13)	0.0542 (7)	
C3	0.7270 (3)	0.4668 (3)	0.12146 (15)	0.0569 (7)	
H3	0.6841	0.3796	0.0915	0.068*	
C4	1.0291 (4)	0.5583 (4)	0.1116 (2)	0.0805 (10)	
H4A	1.1033	0.5076	0.0803	0.097*	
H4B	1.1116	0.6153	0.1457	0.097*	
C5	0.8909 (4)	0.6629 (3)	0.07366 (15)	0.0622 (8)	
C6	0.9218 (4)	0.8021 (4)	0.03314 (19)	0.0838 (10)	
H6A	1.0260	0.8605	0.0563	0.101*	
H6B	0.9517	0.7719	−0.0120	0.101*	
C7	0.7547 (6)	0.8987 (5)	0.0253 (3)	0.1143 (15)	
H7A	0.7502	0.9555	0.0679	0.137*	
H7B	0.7654	0.9740	−0.0108	0.137*	
C8	0.6177 (3)	0.4702 (3)	0.18107 (14)	0.0525 (7)	
C9	0.4688 (4)	0.3726 (3)	0.18314 (15)	0.0593 (7)	
H9	0.4417	0.2997	0.1483	0.071*	
C10	0.3579 (4)	0.3798 (3)	0.23563 (16)	0.0646 (8)	
H10	0.2584	0.3119	0.2360	0.077*	
C11	0.3952 (4)	0.4871 (3)	0.28691 (15)	0.0593 (7)	
C12	0.5476 (4)	0.5848 (3)	0.28669 (16)	0.0657 (8)	
H12	0.5769	0.6556	0.3223	0.079*	
C13	0.6550 (4)	0.5765 (3)	0.23382 (15)	0.0613 (8)	
H13	0.7550	0.6439	0.2335	0.074*	
C14	0.1150 (5)	0.4338 (5)	0.33449 (19)	0.0881 (11)	
H14A	0.0466	0.4550	0.2901	0.132*	
H14B	0.0464	0.4716	0.3699	0.132*	
H14C	0.1330	0.3241	0.3398	0.132*	

### Atomic displacement parameters (Å^2^)

**Table:**

	*U*^11^	*U*^22^	*U*^33^	*U*^12^	*U*^13^	*U*^23^
O1	0.0474 (11)	0.0838 (13)	0.0720 (13)	0.0007 (9)	0.0075 (9)	−0.0005 (10)
O2	0.0686 (13)	0.0824 (14)	0.0777 (14)	−0.0039 (10)	−0.0007 (11)	0.0275 (11)
O3	0.0481 (11)	0.0835 (13)	0.1019 (17)	0.0080 (9)	0.0110 (11)	0.0278 (12)
O4	0.0760 (15)	0.0989 (16)	0.0681 (14)	−0.0059 (12)	0.0208 (11)	0.0058 (11)
C1	0.0532 (16)	0.0636 (15)	0.0458 (14)	−0.0009 (12)	0.0077 (11)	−0.0025 (12)
C2	0.0493 (14)	0.0612 (14)	0.0532 (15)	−0.0073 (11)	0.0109 (12)	−0.0019 (12)
C3	0.0467 (14)	0.0597 (14)	0.0643 (16)	−0.0031 (11)	0.0076 (12)	0.0027 (13)
C4	0.0480 (16)	0.091 (2)	0.103 (2)	−0.0051 (14)	0.0124 (16)	0.0236 (19)
C5	0.0490 (15)	0.0732 (17)	0.0647 (17)	−0.0080 (12)	0.0085 (12)	0.0031 (14)
C6	0.067 (2)	0.098 (2)	0.086 (2)	−0.0250 (17)	0.0082 (17)	0.0274 (19)
C7	0.092 (3)	0.104 (3)	0.144 (4)	−0.017 (2)	0.006 (3)	0.053 (3)
C8	0.0444 (14)	0.0499 (12)	0.0615 (16)	−0.0012 (10)	0.0007 (12)	0.0103 (12)
C9	0.0571 (16)	0.0583 (14)	0.0620 (16)	−0.0110 (12)	0.0051 (13)	0.0006 (13)
C10	0.0543 (15)	0.0695 (16)	0.0691 (18)	−0.0151 (13)	0.0048 (13)	0.0096 (15)
C11	0.0540 (16)	0.0656 (15)	0.0590 (17)	0.0025 (12)	0.0095 (13)	0.0131 (13)
C12	0.0650 (17)	0.0652 (16)	0.0653 (17)	−0.0068 (13)	0.0018 (14)	−0.0034 (14)
C13	0.0507 (15)	0.0629 (15)	0.0696 (18)	−0.0127 (12)	0.0058 (13)	0.0028 (14)
C14	0.068 (2)	0.114 (3)	0.087 (2)	−0.0002 (18)	0.0279 (18)	0.022 (2)

### Geometric parameters (Å, º)

**Table:**

O1—C1	1.198 (3)	C6—H6A	0.9700
O2—C1	1.348 (3)	C6—H6B	0.9700
O2—C7	1.445 (4)	C7—H7A	0.9700
O3—C4	1.411 (4)	C7—H7B	0.9700
O3—C3	1.447 (3)	C8—C9	1.374 (4)
O4—C11	1.371 (4)	C8—C13	1.381 (4)
O4—C14	1.418 (4)	C9—C10	1.383 (4)
C1—C2	1.448 (4)	C9—H9	0.9300
C2—C5	1.326 (4)	C10—C11	1.366 (4)
C2—C3	1.501 (4)	C10—H10	0.9300
C3—C8	1.490 (4)	C11—C12	1.390 (4)
C3—H3	0.9800	C12—C13	1.372 (4)
C4—C5	1.476 (4)	C12—H12	0.9300
C4—H4A	0.9700	C13—H13	0.9300
C4—H4B	0.9700	C14—H14A	0.9600
C5—C6	1.472 (4)	C14—H14B	0.9600
C6—C7	1.461 (5)	C14—H14C	0.9600
			
C1—O2—C7	118.5 (2)	O2—C7—C6	115.2 (3)
C4—O3—C3	111.1 (2)	O2—C7—H7A	108.5
C11—O4—C14	117.3 (3)	C6—C7—H7A	108.5
O1—C1—O2	118.2 (2)	O2—C7—H7B	108.5
O1—C1—C2	125.4 (3)	C6—C7—H7B	108.5
O2—C1—C2	116.4 (2)	H7A—C7—H7B	107.5
C5—C2—C1	122.7 (3)	C9—C8—C13	117.7 (3)
C5—C2—C3	111.0 (2)	C9—C8—C3	120.6 (2)
C1—C2—C3	126.3 (2)	C13—C8—C3	121.6 (2)
O3—C3—C8	111.6 (2)	C8—C9—C10	121.8 (3)
O3—C3—C2	102.55 (19)	C8—C9—H9	119.1
C8—C3—C2	115.9 (2)	C10—C9—H9	119.1
O3—C3—H3	108.9	C11—C10—C9	119.7 (3)
C8—C3—H3	108.9	C11—C10—H10	120.1
C2—C3—H3	108.9	C9—C10—H10	120.1
O3—C4—C5	105.4 (2)	C10—C11—O4	124.7 (3)
O3—C4—H4A	110.7	C10—C11—C12	119.4 (3)
C5—C4—H4A	110.7	O4—C11—C12	115.9 (3)
O3—C4—H4B	110.7	C13—C12—C11	119.8 (3)
C5—C4—H4B	110.7	C13—C12—H12	120.1
H4A—C4—H4B	108.8	C11—C12—H12	120.1
C2—C5—C6	121.4 (3)	C12—C13—C8	121.5 (3)
C2—C5—C4	109.5 (3)	C12—C13—H13	119.3
C6—C5—C4	129.1 (2)	C8—C13—H13	119.3
C7—C6—C5	110.0 (3)	O4—C14—H14A	109.5
C7—C6—H6A	109.7	O4—C14—H14B	109.5
C5—C6—H6A	109.7	H14A—C14—H14B	109.5
C7—C6—H6B	109.7	O4—C14—H14C	109.5
C5—C6—H6B	109.7	H14A—C14—H14C	109.5
H6A—C6—H6B	108.2	H14B—C14—H14C	109.5

## References

[bb1] Bruker (2002). *SMART*, *SAINT* and *SADABS* Bruker AXS Inc., Madison, Wisconsin, USA.

[bb2] Sheldrick, G. M. (2008). *Acta Cryst.* A**64**, 112–122. 10.1107/S0108767307043930 18156677

[bb3] Wang, T. T., Liu, J., Zhong, H. Y., Chen, H., Lv, Z. L., Zhang, Y. K., Zhang, M. F., Geng, D. P., Niu, C. J., Li, Y. M. & Li, K. (2011). *Bioorg. Med. Chem. Lett.* **21**, 3381–3383. 10.1016/j.bmcl.2011.04.003 21515044

